# Computational and Mathematical Methods in Cardiovascular Diseases

**DOI:** 10.1155/2017/4205735

**Published:** 2017-03-19

**Authors:** Elena G. Tolkacheva, Xiaopeng Zhao, Sharon Zlochiver, Yoichiro Mori

**Affiliations:** ^1^Department of Biomedical Engineering, University of Minnesota, Minneapolis, MN, USA; ^2^Department of Mechanical, Aerospace, and Biomedical Engineering, University of Tennessee, Knoxville, TN, USA; ^3^Department of Biomedical Engineering, Tel-Aviv University, Tel Aviv, Israel; ^4^Department of Mathematics, University of Minnesota, Minneapolis, MN, USA

 Cardiovascular diseases are a leading cause of death in the world. Computational and mathematical methods provide a useful tool to better understand heart and vessel problems. This special issue focuses on various computational and mathematical methods to model cardiac disorders, to understand the existing mathematical challenges, to explore new directions in modeling of cardiovascular dynamics and cardiac rhythm abnormalities, and to develop cardiac related mathematical applications in clinical and emergency situations.

Several key aspects become the main targets for discussion in the current special issue.

Several authors presented their research aiming to use computational approaches to elucidate the underlying electrophysiological mechanisms of different cardiac diseases leading to increased arrhythmogeneity in the heart. One area of research includes investigating the roles of hyperkalemia and calcium handling components played in the genesis of alternans in ischemia at the cellular level. The results show that hyperkalaemic conditions reduced cell excitability and delayed recovery from inactivation of depolarization currents, thus leading to alternans formation. In addition, sarcoplasmic reticulum calcium-ATPase (SERCA2a) function decreased in ischemia, thus resulting in intracellular Ca (Cai) alternans of small magnitude. Finally, a strong Na^+^-Ca^2+^ exchange current (INCX) increased the magnitude of Cai alternans, leading to APD alternans through excitation-contraction coupling. Another research aimed to investigate the combined role of Ca2+/calmodulin-dependent protein kinase II (CaMKII) and *β*-adrenergic signaling pathway in regulating early afterdepolarization. Early afterdepolarization (EAD) has tremendous relevance for the onset of arrhythmogenesis. Simulations of this kind provide insights on therapy strategies to treat EADs related arrhythmogenesis. Finally, there is an interest to determine the potential antiarrhythmic effects of the green tea catechin Epigallocatechin-3-Gallate (E3G), by employing numerical simulations of cellular and cable models using atrial, Purkinje, and ventricular kinetics. The authors tested the effects of the drug on action potential properties and conduction velocity in the settings of two Na+ channel gain of function mutations that are correlated with hyperexcitability and ectopic premature contractions. They concluded that 30 *μ*M E3G reduces and suppresses cellular electrical abnormalities that are associated with those mutations.

Another important aspect was the development and application of different methods and techniques to various cardiovascular recordings in order to differentiate between diseased and healthy states. Some studies propose to investigate the level of temporal dependency in cardiovascular time series using a copula method. The technique was applied to healthy aging rats as well as aging rats with early developed hypertension. The study shows that copulas and conditional entropy can reveal dependency of streams, to their number and to type. The authors highlighted that antiparallel streams play an important role between systolic blood pressure and its pulse interval. Another approach is to use a 3D modeling for hemodynamic analysis of fluid structure interaction in intima and adventitia. Combining CT, IVUS, and biplane X-ray angiogram images, the authors calculated deformation of intima and adventitia by catheter insertion and generated vessel bifurcations. Limitations and future directions were also discussed. Finally, a hybrid classification system using the Rough Set and Relief (RSRF) method for diagnosis of heart diseases can be developed. Numerical experiments were conducted using the Statlog data. Results that reported on 10-fold cross validation demonstrate a high classification accuracy.

Several authors used computational approach to study various biomechanical properties and characteristics of the various diseased hearts. Some studies evaluate the pumping efficacy of a left ventricular assist device according to cannulation site in heart failure with valvular regurgitation. They aimed to analyze the contribution of a left ventricular assist device (LVAD) to mitral and aortic valve regurgitation for the following cannulation sites: from the LA to the aorta (LAAO) and from the LV to the aorta (LVAO) under different conditions. The results showed that LVAD with LAAO cannulation is appropriate for recovery of the mitral valve regurgitation heart, and the LVAD with LVAO cannulation is appropriate for treating the aortic valve regurgitation heart. Another approach is to investigate the relationship between endoleak formation and the surrounding pathological flow fields. Endoleak formation is a major complication for abdominal aortic aneurysm patients. Using computational fluid dynamics and image processing techniques, the authors reconstructed 6 patient specific models to study possible endoleak formation in a location with high local wall stress. The developed models may be adapted for other human cardiovascular surgeries, such as cardiopulmonary bypass, where detailed patient-specific hemodynamics is necessary.

*Elena G. Tolkacheva* *Xiaopeng Zhao* *Sharon Zlochiver* *Yoichiro Mori*

